# Obesity among type 2 diabetes mellitus at Sidama Region, Southern Ethiopia

**DOI:** 10.1371/journal.pone.0266716

**Published:** 2022-04-14

**Authors:** Temesgen Bizuayehu, Tadesse Menjetta, Metsihet Mohammed

**Affiliations:** 1 School of Medical Laboratory Science, College of Medicine and Health Sciences, Hawassa University, Hawassa, Ethiopia; 2 Department of Laboratory, Hawassa University Comprehensive Specialized Hospital, Hawassa, Ethiopia; Moi University College of Health Sciences, KENYA

## Abstract

**Background:**

Type 2 diabetes is becoming highly prevalent worldwide and it is one of the leading causes of mortality. The cause of mortality among these patients is mostly related to the dominant presence of modifiable cardiovascular risk factors such as obesity. The aim of the current study is therefore to determine the prevalence of obesity and its associated factors among patients with type 2 diabetes mellitus at Sidama region, Ethiopia.

**Method:**

**I**nstitution-based cross-sectional study design was implemented to determine the prevalence of obesity and its associated factor among patients with type two diabetes at Hawassa University Comprehensive Specialized Hospital and Yirgalem General Hospital from October 16 2018 to December 21, 2018. A simple random sampling technique was implemented to select 314 study participants. After obtaining consent, different patients’ related data were collected using a questionnaire. Patients’ records were also reviewed. 4ml of the blood sample was collected from each study participant and analyzed for lipid profile test. Blood glucose level was done using COBAS INTEGRA 6000. A binary logistic regression was used to assess factors that have an association with obesity. A P-value of <0.05 was considered statistically significant.

**Result:**

The majority of the study participants (67.2%) were male and 61.8% of the study participants were aged >45years. The overall prevalence of overweight and obesity among the study participants was 36.3% and 18.8% respectively. About 41% of the study participants have a normal BMI. Females were more obese (28.2% Vs 14.2%) than males and BMI stratification by sex was statistically significant (P = 0.02). Sex (AOR = 3.0, CI = 1.6–5.7, P-Value = 0.001) and TG (AOR = 3.6, CI = 1.6–8.3, P-Value = 0.003) are factors that were independently associated with obesity among type 2 DM patients.

**Conclusion:**

Overweight and obesity among type two diabetic patients were prevalent. In addition, obesity and overweight disorder are common among T2DM and gender and triglycerides levels were associated with obesity.

## Background

Diabetes has been one of the enormous global public health problems hurting a large number of people. Type 2 diabetes is the major type of diabetes mellitus (DM) related to a different form of cardiovascular complication, and obesity is a potentially modifiable risk factor for T2DM. Obesity-triggered cardiovascular complications are related to a high number of deaths worldwide [1].

The prevalence of diabetes mellitus among the Ethiopian community ranges from 2% in the Tigray region to 14% in Dire Dewa city [2]. A meta-analysis study indicated that the pooled prevalence of DM is 6.5%. The prevalence of DM in health facilities is 8% while in the community it ranges from 1.9% [3] in the Southern region of Ethiopia to 12.4% in Hawassa Zuria [4]. The etiology of obesity is multifactorial, exceedingly complicated involving the interaction of different factors such as behavioral, environmental, physiological, genetic, social, and economic factors. Both T2DM and obesity are related to different diseases and raises the risk of cardiovascular complication.

Low cardiac output, poorer systolic function, and ventricular wall thickness are some of the burdens of obesity to the cardiovascular system [5]. Its influence may also be extended to affect coronary risk indirectly through its effect on related co-morbidities. These co-morbidities include but are not limited to increased blood pressure, Insulin resistance, lipid derangements, and inflammation [6–8]. A higher BMI among T2DM than a healthy individual is a well-known truth that shows a solid epidemiological correlation between the development of T2DM and obesity.

The attention given to the importance of obesity in the clinic population of patients with T2DM is not satisfactory. Understanding Obesity among these patients has an important interest as it is an independent risk factor for CVD and because it has a strong correlation with metabolic syndrome.

The aim of this study is therefore to determine the prevalence of obesity and to assess its associated factor among T2DM patients at Hawassa University Comprehensive Specialized Hospital (HUCSH) and Yirgalem General Hospital (YGH), Sidama region, Southern Ethiopia.

## Method and materials

### Study design, area, and period

A cross-sectional study was conducted at HUCSH and YGH from October 16-December21, 2018. Currently, HUCSH is the only specialized hospital in the region with more than four hundred beds and offers services at the general and specialty level. It has also established a diabetic clinic. YGH is one of the oldest hospitals found in the Sidama region of Ethiopia. It is located at Yirgalem Town, 45 km South of Hawassa city, the capital of the region. T2DM patients at HUCSH and YGH diabetic clinic are considered as a source population. T2DM patients visiting YGH and HUCSH diabetic clinics during the study period fulfilling the inclusion criteria were considered as study participants.

### Ethics approval

Ethical approval was obtained from the Institutional Review Board of the College of Health Science, Jimma University. All study participants were given adequate information regarding the risk, benefit, and confidentiality of the study. They were also informed that they can withdraw from the study at any time. Then, informed written consent was obtained from each study participant.

### Sample size and sampling technique

A single population proportion formula was used to calculate the total sample size. By using the systematic random sampling method, a total of 314 T2DM participants were included from a total of 950 T2DM patients who were on treatment follow-up in YGH and HUCSH. The total sample size was proportionally allocated to YGH (83) and HUCSH (231) to select the required sample size. Those patients who were on blood lipid-lowering medication, pregnant women, and contraceptive users were excluded from the study.

### Socio-demographic, clinical and other data collection

Socio-demographic, clinical and anthropometric data were gathered by trained data collectors utilizing a structured questionnaire through face-to-face interviews and physical measurement of height, weight and blood pressure using standardized techniques and calibrated equipment. A detailed review of medical records was performed and the WHO guideline manual [9] was followed for anthropometric data. Weight and height were measured with participants standing without shoes and wearing light clothing. Participants were standing upright with the head, shoulder, buttock, lower limb and heel of the foot touching the height board for height measurement. Body mass index (BMI) was calculated by dividing weight by height square and expressed as kilogram per meter square (Kg/M^2^). Systolic and diastolic blood pressure was measured by a mercury-based sphygmomanometer after the participants had rested for more than 10 minutes. The precision of the measurement was checked by taking two measurements two minutes apart and by taking the average of the two measurements. For those study participants with systolic blood pressure (SBP) of ≥ 140mmHg and diastolic blood pressure (DBP) of ≥ 90 mmHg, BP was repeated and finally, the mean of the two measurements was taken.

### Blood specimen collection and sample analysis

First, after overnight fasting, 4ml of venous blood was collected from each participant by a gel separator tube. Then, after the collected blood was allowed to completely clot, it was centrifuged at 3000 revolutions per minute to separate the serum part. Finally, COBAS INTEGRA 6000 was used to analyze the serum for high-density lipoprotein cholesterol (HDL-C), low-density lipoprotein cholesterol (LDL-C), Triglycerides (TGs), total cholesterol (CHOL-T), and fasting blood sugar (FBS). A direct enzymatic method was used to measure lipid profile tests.

### Data quality management and statistical analysis

The questionnaire was prepared in English translated to Amharic language then back to English language. All data collectors were trained for one day on an overview of the assessment and the objectives of the research by the principal investigators before the beginning of any data collection. All the data were checked daily for consistency, accuracy and completeness visually. Standardized procedures were strictly followed during the blood sample collection, storage and analysis. The quality of the test, the proper functioning of the instrument and its technical performance and the quality of laboratory reagent were maintained by running a quality control sample and the run was repeated for results falling outside the reference interval.

Data were entered into a Statistical Package for Social science software (version 20.0 SPSS Inc. USA) for statistical data analysis. Descriptive statistics like frequency and percentage were computed to describe the data. Then, a Chi-square test was performed to examine and compare between the study groups. Besides, the difference in mean of the study groups was evaluated using a student t-test. To identify factors associated with obesity, multivariable logistic regression was used after identifying candidate variables by bivariate analysis. Odds ratio (OR) and respective 95% confidence interval (CI) was used to estimate the effect size for the association and a P-value of<0.05 was considered statistically significant.

### Operational definition

BMI, expressed in kg/m^2^, was categorized into a major four categories: BMI <18.5 (underweight), BMI between 18.5–24.9 (normal weight), BMI between 25.0–29.9 (overweight) and BMI ≥30.0 (Obese). Obesity was further categorized as grade I (BMI = 30.0–34.9), Grade II (BMI = 35.0–39.9), and grade III (BMI ≥40) respectively. A Waist-to-Height ratio (WhtR) cut-off value of <0.6 and ≥0.6 which was effective in predicting cardiovascular disease risk in T2DM patients, was applied in this study [10].

## Result

### Socio-demographic, clinical and other characteristics of the study participants

A total of 314 study participants were included in this study and the majority of the participants were males. 89.8% do not drink alcohol, 89.5% do not smoke and 68.2% do not do regular exercise. 79.0% of the study subjects had diabetes mellitus duration of less than 10 years. 30.9% of the study participants had a WhtR of ≥0.6 (Table 1).

**Table 1:** Socio-demographic and clinical characteristics of the study participants stratified by BMI in patients with T2DM at HUCSH and YGH from Oct 16 to Dec. 21, 2018.

variable	Total	Underweight	Normal	Overweight	Obese	P-value
Age						
<45	120(38.2)	5(4.2)	58(48.3)	39(32.5)	18(15.0)	0.01*
45–60	150(47.8)	1(0.7)	58(38.7)	58(38.7)	33(22.0)	
>60	44(14.0)	5(11.4)	14(31.8)	17(38.6)	8(18.2)	
Sex						
Male	211(67.2)	6(2.8)	92(43.6)	83(39.3)	30(14.2)	0.02*
Female	103(32.8)	5(4.9)	38(36.9)	31(30.1)	29(28.2)	
Residence						
Urban	159(50.6)	6(3.8)	55(34.6)	64(40.3)	34(21.4)	0.2
Rural	155(49.4)	5(3.2)	75(48.4)	50(32.3)	25(16.1)	
Educational status						
No formal education	63(20.1)	6(9.5)	28(44.4)	19(30.2)	10(15.9)	
1^0^	124(39.5)	1(0.8)	48(38.7)	45(36.3)	30(24.2)	0.03*
≥2^0^	127(40.4)	4(3.1)	54(42.5)	50(39.4)	19(15.0)	
Alcohol drink						
No	282(89.8)	9(3.2)	123(43.6)	95(33.7)	55(19.5)	0.02*
Yes	32(10.2)	2(6.2)	7(21.9)	19(59.4)	4(12.5)	
Smoking						
No	281(89.5)	9(3.2)	121(43.1)	95(33.8)	56(19.9)	0.03*
Yes	33(10.5)	2(6.1)	9(27.3)	19(57.6)	3(9.1)	
Regular exercise						
No	214(68.2)	8(3.7)	85(39.7)	76(35.5)	45(21.0)	0.5
Yes	100(31.8)	3(3.0)	45(45.0)	38(38.0)	14(14.0)	
Duration of DM						
≤10	248(79.0)	8(3.2)	104(41.9)	93(37.5)	43(17.3)	
>10	66(21.0)	3(4.5)	26(39.4)	21(31.8)	16(24.2)	0.5
Family history of DM						
No	259(82.5)	5(1.9)	108(41.7)	96(37.1)	50(19.3)	
Yes	55(17.5)	6(10.9)	22(40.0)	18(32.7)	9(16.4)	0.01*
WhtR						
<0.6	217(69.1)	10(4.6)	126(58.1)	64(29.5)	17(7.8)	
≥0.6	97(30.9)	1(1.0)	4(4.1)	50(51.5)	42(43.3)	<0.001*

*P<0.05; WhtR, Weight to height Ratio.

The prevalence of overweight and obesity among the study subjects was 36.3% and 18.8% respectively. 41.4% of the participants have normal BMI. 39.3% males and 30.1% females are overweight, and females are more obese (28.2% Vs 14.2%) than males and BMI stratification by sex was statistically significant (P = 0.02). 59.4% of the study participants who drink alcohol were overweight and 12.5% of them were obese. 35.5% of the study participants who do not practice regular exercise were overweight 21% of them were obese (Table 1).

56.1% of overweight and 57.6% of obese patients were urban dwellers. The majority, (43.9% and 32.2%) of both overweight and obese study participants had 2^0^ and above education level. 76.3%, 72.9%, 84.7, and 71.2% of obese patients were those who do not practice regular exercise, had a diabetic duration of ≤10 years, do not have a family history of DM, and had a WhtR of ≥0.6 respectively.

#### FBS, BP, and lipid profiles across the four different bands of BMI among T2DM patients

The majority, (91.5%) of obese patients had an FBS of >110mg/dl and the distribution of FBS across different BMI strata is statistically significant (P = 0.002). 47.5% and 40.7% of obese study participants had elevated SBP and DBP respectively. The median (IQR) of triglyceride, cholesterol and HDL-C among the study participants are 194mg/dl (139.5–246.0), 180.5mg/dl(153.0–208.3) and 48mg/dl (38.0–60.3) respectively. The median (IQR) of triglyceride (247.0, 197.0–277.0), cholesterol (187.0, 156.8–215.3) and LDL-C (103, 70.0–120.0) among Obese study participants are higher than the other three strata (underweight, normal and overweight) and the distribution of lipid profile among these strata was statistically significant except for TG (Table 2).

**Table 2:** FBS, blood pressure, and lipid profiles across the four different bands of BMI in patients with T2DM at HUCSH and YGH from Oct 16 to. Dec. 21, 2018.

Variables	Total	Underweight	Normal	Overweight	Obese
		n = 11	n = 130	n = 114	n = 59
FBS					
≤110 Mg/dl	39(12.4)	5(45.5)	23(17.7)	6(5.2)	5(8.5)*
>110Mg/dl	275(87.6)	6(54.5)	107(82.3)	108(94.8)	54(91.5)
SBP					
Normal	197(62.7)	6(54.5)	95(73.1)	65(57.0)	31(52.5)*
Abnormal	117(37.3)	5(45.5)	35(26.9)	49(43.0)	28(47.5)
DBP					
Normal	226(72.0)	10(90.9)	106(81.5)	75(65.8)	35(59.3)
Abnormal	88(28.0)	1(9.1)	24(18.5)	39(34.2)	24(40.7) *
Dyslipidemia					
No	38(12.1)	5(13.2)	23(60.5)	9(23.7)	1(1.7)
Yes	276(87.9)	6(2.2)	107(38.8)	105(38.0)	58(98.3) *
Cholesterol (Median, IQR)	180.5 (153.0–208.3)	151.0 (104.0–195.0)	92.0 (69.5–105.0)	182.0 (157.0–182.5)	187.0 (156.8–215.3) *
TG (Median, IQR)	194.5 (139.5–246.0)	129.0 (114.0–141.0)	162.0 (128.5–204.0)	202.0 (147.0–249.3)	247.0 (197.0–277.0)
HDL (Median, IQR)	48 (38.0–60.3)	42.0 (38.0–62.0)	46.0 (35.8–55.3)	48.5 (38.5–62.3)	50 (39.0–65.0) *
LDL (Median, IQR)		59 (38.0–114.0)	92.0 (69.5–105.0)	89.0 (75.0–116.3)	103 (70.0–120.0) *

*P<0.05; DBP, Diastolic blood pressure; DDM, Duration of diabetes Mellitus; FBS, fasting blood sugar; HDL-C, High-density lipoprotein; IQR, Interquartile range; LDL-C, Low-density lipoprotein; NFE, No formal education; SBP, systolic blood pressure; TG, triglycerides.

#### Prevalence of different categories of obesity

**Fig 1:**
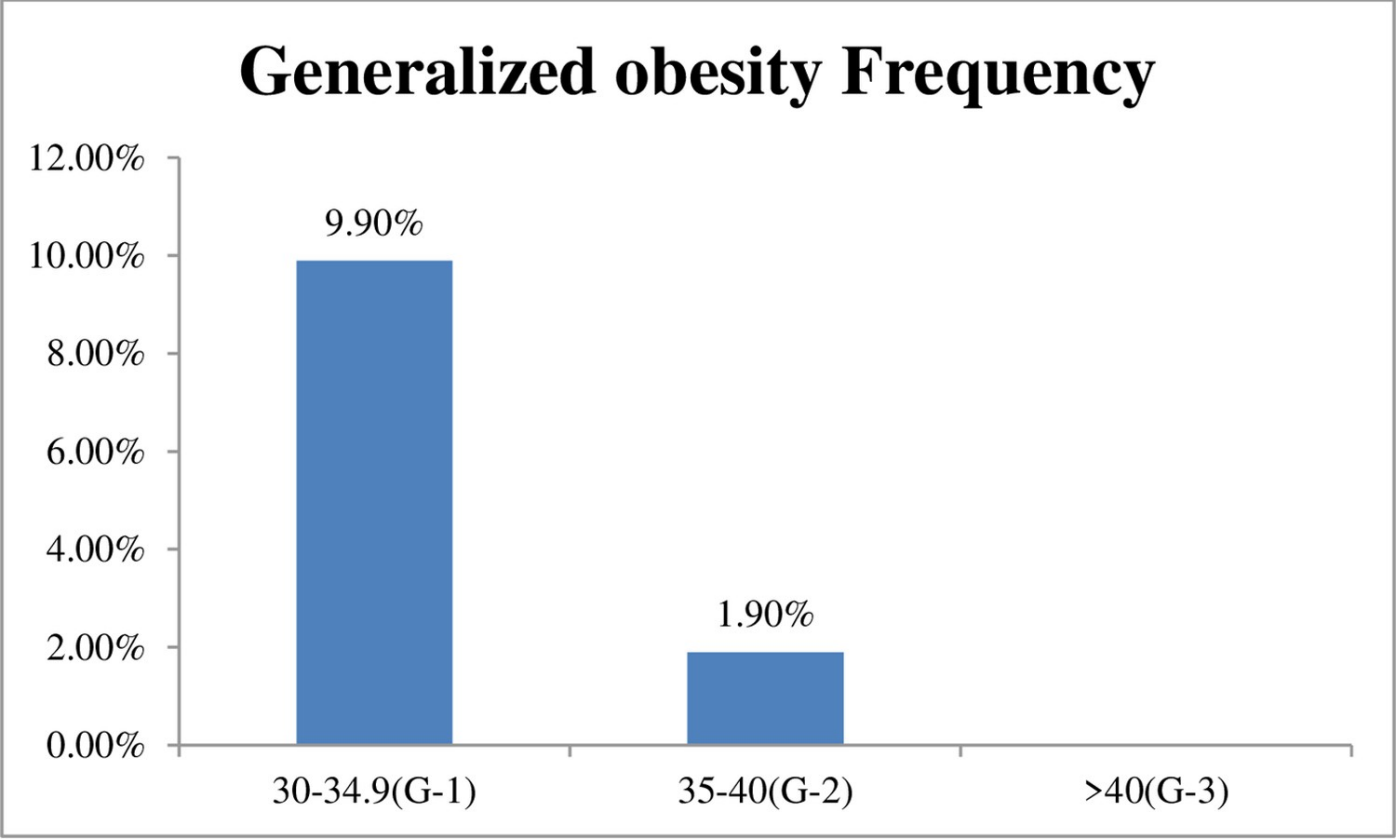
The different grades of obesity among the study participants.

As it is shown in Fig 1, 9.9% of the study participants had grade I obesity (BMI = 30–34.9 kg/m^2^) while 1.9% of the study participants had grade II (BMI = 35–40 kg/m^2^) (Fig 1).

#### Factors associated with obesity among T2DM study participants

Sex, smoking status, regular exercise, uric acid level, SBP, DBP, Cholesterol level, TGs, LDL-C level dyslipidemia were variables with a P-Value of <0.2 and inserted into Multivariate logistic regression. Sex (AOR = 3.0, CI = 1.6–5.7, P = 0.001) and TG (AOR = 3.6, CI = 1.6–8.3, P = 0.003) are variables that were independently associated with obesity among type 2 DM patients (Table 3).

**Table 3:** Factors associated with obesity among T2DM patients at HUCSH and YGH from Oct 16 to Dec. 21, 2018.

Variables	Category	Obesity	COR(CI)	P-Value	AOR(CI)	P-Value
		Yes				
sex	Male	30(14.2)	1		1	
	Female	29(28.2)	2.4(1.3–4.3)	0.004*	3.0(1.6–5.7)	0.001**
Age	<45	18(15.0)	1			
	≥45	41(21.1)	1.5(0.8–2.8)	0.2		
Residence	Urban	34(21.4)	1.4(0.8–2.5)	0.2		
	Rural	25(16.1)	1			
Educational level	NFE	10(15.9)	0.7(0.3–1.9)	0.5		
	Primary	30(24.2)	1.2(0.5–2.8)	0.7		
	Secondary	10(11.9)	0.5(0.2–1.4)	1.8		
	Tertiary	9(20.9)	1			
Alcohol drink	No	55(19.5)	1			
	Yes	4(12.5)	0.6(0.2–1.8)	0.3		
smoking	No	56(19.9)	1		1	
	Yes	3(9.1)	2.5(0.7–8.5)	0.1*	3.0(0.8–10.7)	0.08
Regular exercise	No	45(21.0)	1.6(0.9–3.1)	0.1*	0.7(0.3–1.4)	0.7
	Yes	14(14.0)	1		1	
Uric acid level	Normal	28(13.5)	1		1	
	Hyperuricemia	31(29.2)	2.7(1.5–4.7)	0.001*	1.2(0.6–1.6)	0.6
DDM	≤10	43(17.3)	1			
	>10	16(24.2)	0.7(0.3–1.3)	0.2		
FHDM	No	50(19.3)	1			
	Yes	9(16.4)	0.8(0.4–1.8)	0.6		
SBP	Normal	31(15.7)	1		1	
	Abnormal	28(23.9)	1.7(1.0–3.0)	0.07*	0.8(0.3–1.9)	0.6
DBP	Normal	33(15.8)	1	1		
	Abnormal	26(24.8)	1.7(1.0–3.0)	0.07*	1.8(0.8–4.4)	0.2
FBS	<110 mg/dl	5(12.8)	1			
	≥110 mg/dl	54(19.6)	1.5(0.7–3.2)	0.3		
Cholesterol	<200 mg/dl	30(14.3)	1		1	
	≥200 mg/dl	29(27.9)	2.3(1.3–4.1)	0.004*	1.3(0.6–3.1)	0.6
TG level	<200 mg/dl	16(9.6)	1		1	
	≥200mg/dl	43(29.3)	3.9(2.1–7.3)	<0.001*	3.6(1.6–8.3)	0.003**
HDL-C Level	< 45 mg/dl	33(17.3)	1.2(0.7–2.3)	0.4		
	≥45 mg/dl	26(21.1)	1			
LDL -C	<100mg/dl	28(15.1)	1		1	
	≥100 mg/dl	31(24.2)	1.8(1.0–3.2)	0.04*	1.5(0.6–3.3)	0.4
Dyslipidemia	No	1(2.6)	1		1	
	Yes	58(21.0)	9.8(1.3–73.3)	0.03*	3.1(0.4–25.5)	0.3

P<0.2; P<0.05; AOR, Adjusted odds ratio; CI, Confidence interval; COR, Crude odds ratio; DBP, Diastolic blood pressure; DDM, Duration of diabetes Mellitus; FBS, fasting blood sugar; HDL-C, High-density lipoprotein; LDL-C, Low-density lipoprotein; NFE, No formal education; SBP, systolic blood pressure; TG, triglycerides.

## Discussion

Obesity amongT2DM has become an enormous public health problem and a bigger health crisis than hunger and the leading cause of death and disabilities around the world with the burden expected to increase through time. It has long been established as one of the independent risk factors for CVD [11] and is associated with a different form of CVD [12–14]. The main findings of this research are: 1) the prevalence of obesity among T2DM which is 18.8% with 9.9% grade I obesity and 1.9% grade II obesity 2) disparities of obesity by gender 3) factors associated with obesity (TG (AOR = 3.6, CI = 1.6–8.3, P = 0.003).

The overall prevalence of obesity among the study subjects was 18.8% which is higher than the study from southwest Ethiopia (6.9%) [15], Southern Ethiopia Hosanna (4.5%) [16], UK (16.6%) [17], China (7.14%) [18] and lower than the study done in Northern Ethiopia, Tigray region (53.2%) [19], Nigeria (27.4%) [20], Liverpool, UK (52%) [17], Yemen (28.8%) [21], Saudi Arabia (38.2%) [22], Turkey (59%) [23] and Al-Khobar (39.9%) [24]. Applied obesity criteria (BMI, WC, or waist/hip ratio) Physical activity differences, sleep differences, diet differences and racial and ethnic disparities may be the triggering factor for the difference in the prevalence of obesity among different studies [25]. Country specified obesity criteria and obesity thresholds should be adapted for each country. The difference among studies could also be due to the difference in the research methodologies used by the investigators.

About 36.3% of the study participants were overweight which was lower than the study from the Northern Ethiopia, Tigray region (40.8%) [19], UK (55.3%) [17] and China (38.77%) [18] but higher than the study done in Southwest Ethiopia (29.3%) [15] and Hosanna Town, Southern Ethiopia Hosanna (31.3%) [16]. Around 55.1% of the study participants were either overweight or obese which is higher than the study done in Southwest Ethiopia (36.2%) [15], Addis Ababa Ethiopia (46.4%) [26] and Hosanna (35.9%) [16] but less than the study from Liverpool (86%) [17]. Among patients with age ≥45 years, 36.9% of them were either overweight or obese which is lower than the study from the United States where 85.2% of the study participants with T2DM were either overweight or obese [27].

Around 41.4% of them had normal BMI which is in contrast with the study from Saudi Arabia [22]. Females were more obese (28.2%) than males (14.2%) which is in line with the study done in Nigeria [20], Turkey [23], Saudi Arabia [22], Yemen [21] and the UK [17] but in contrast with some other studies [28]. The higher obesity among females is probably due to short intervals between pregnancies, use of oral contraceptive pills, menopause-related hormonal imbalance, dietary habits, and sedentary lifestyle [29]. Women with T2DM have a 3 times greater risk of obesity than men. (AOR = 3.0, CI = 1.6–5.7, P = 0.001) which is in line with the study from Turkey [23] and Yemen [21].

TG is the only variable that was independently associated (AOR = 3.6, CI = 1.6–8.3, P = 0.003) with obesity among T2DM patients. T2DM patients in different UK clinics had a higher TG level than non-obese patients [17]. Similarly, a study from China found that overweight or obesity was associated with an increased likelihood of co-morbid dyslipidemia [18].

BP and FBS levels were higher among overweight and obese study participants. BMI was strongly associated with SBP and BP among T2DM and most of the hypertensive patients were happened to be obese. Therefore, hypertension is one of the most common obesity-related disorders [30, 31]. Cardiovascular risk events such as hyperglycemia elevated BP, and abnormal lipid profiles were prevalent in the current study. As BMI increases, insulin resistance will also increase which results in hyperglycemia in the body.

Studies observed that individuals with T2DM have two to three folds greater risk of cardiovascular events compared with subjects without diabetes, and CVD is responsible for almost 80% of the mortality in T2DM [32] though the influence of these events is not proportional. Lowering blood pressure and improving the lipid profile leads to a greater reduction in CVD risk than lowering plasma glucose in T2DM. It is also obvious that obesity is predictive of future coronary disease events among T2DM and succeeding in losing weight often enjoys modest improvements in glycemic control and CVD risk profiles.

### Limitation of the study

Its cross-sectional nature and small sample size make this study not to be generalizable to the larger population. The other limitation of the study is that obesity was only defined by BMI and triglyceride measurement may be confounded by other variables. The effect of some anti-diabetic medication was not also assessed. A large-scale study with an upgraded method is needed to study the relationship between obesity and T2DM.

## Conclusion

Generally, from the recent study, we can conclude that obesity and overweight disorder are common among T2DM and gender and triglycerides levels were associated with obesity.

## Supporting information

S1 File (DOCX)
