# Supplementary material for: Obesity among type 2 diabetes mellitus at Sidama Region, Southern Ethiopia
Source: PLoS One. 2022 Apr 14;17(4):e0266716. doi: 10.1371/journal.pone.0266716 (PMC9009681; doi:10.1371/journal.pone.0266716)
Supplement: S1 File — (DOCX) [file pone.0266716.s001.docx]

**Questionnaire**

Questionnaire for the demographic characteristics and investigation of Obesity among type 2 Diabetes Mellitus at Sidama Region, Southern Ethiopia

**Socio-demographic data**

1. Sex
   1. Male
   2. Female
2. Age (in years): _________
3. Residence
   1. Urban
   2. Rural
4. Educational status
   1. Illiterate
   2. Primary
   3. Secondary
   4. Tertiary
5. Marital status
   1. Single
   2. Married
   3. Divorced
   4. Widowed
6. Occupation
   1. Farmer
   2. .employed
   3. Daily labourer
   4. House wife
   5. Others specify_________
7. Monthly income in (ETB)_______________
8. Mode of transportation
   1. Walking or bicycle
   2. Motorized vehicle
9. Work type
   1. Manual
   2. Office
   3. Vigorous

**Part II: Quationnaire for clinical data**

1. Do you have family history of DM?
   1. Yes
   2. No
2. Do you smoke?
   1. Yes
   2. No
3. If the answer is yes for the above questions (#11), how often do you smoke?
   1. Never smoking
   2. Current smoker
   3. Former smoker
4. Do you drink alcohol?
   1. Yes
   2. No
5. If the answer is yes for the above questions (#13), how often do you drink?
   1. Never
   2. Once per week
   3. ≥ once per week
   4. daily
6. Duration of DM_________
7. Are you pregnant? (for female participant only)
   1. Yes
   2. No
8. Physical activity
   1. Low
   2. Moderate
   3. High
9. Are you doing regular physical exercise?
   1. Yes
   2. No
10. On a typical week, how much time do you spend in total on moderate and vigorous physical
    activities where your heartbeat increases and you breathe faster? Only include activities that lasted at least 10 minutes at a time.
11. Less than ½ an hour (less than 30 minutes)
12. ½ an hour - 1 ½ hours (30-90 minutes)
13. 1 ½ - 2 ½ hours (90-150 minutes)
14. 2 ½ - 5 hours (150-300 minutes)
15. More than 5 hours (more than 300 minutes)
16. How much of the time that you spend on physical activities in a typical week, which you
    indicated above, do you spend in total on vigorous physical activities? This includes activities that get your heart racing, make you sweat and leave you so short of breath that speaking becomes difficult. Only include activities that lasted at least 10 minutes at a time.
17. Less than ½ an hour (less than 30 minutes)
18. ½ an hour - 1 hours (30-60 minutes)
19. 1 - 1 ½ hours (60-90 minutes)
20. 1 ½ - 2 ½ hours (90-150 minutes)
21. More than 2 ½ hours (more than 150 minutes)

**PART III: Anthropometric measurnment**

1. Height (in meter) __________________
2. Weight (in Kg) __________________
3. BMI (in Kg/m^2^) __________________
4. Blood pressure( SBP/DBP in mmHg) __________________
